# Supplementary material for: Misophonia impact questionnaire (MIQ), tinnitus impact questionnaire (TIQ), and hyperacusis impact questionnaire (HIQ): Factor analysis, test-retest reliability, and minimum detectable change using a non-clinical population
Source: PLoS One. 2025 Jun 5;20(6):e0324726. doi: 10.1371/journal.pone.0324726 (PMC12140247; doi:10.1371/journal.pone.0324726)
Supplement: S1 Appendix — (DOCX) [file pone.0324726.s001.docx]

**S1 Appendix :** Misophonia Impact Questionnaire

| Please answer each item to the best of your ability as close to your experience as possible.  Over the last 2 weeks, how often would you say the following has occurred because of your intolerance to certain sounds related to eating, chewing gum, lip smacking, mouth noises, sniffling, breathing, clicking, and tapping? | | | | |
| --- | --- | --- | --- | --- |
| 1. Feeling anxious | 0-1 day | 2-6 days | 7-10 days | 11-14 days |
| 1. Unable to distract yourself from certain sounds | 0-1 day | 2-6 days | 7-10 days | 11-14 days |
| 1. Experiencing difficulties in your relationships with family members or friends | 0-1 day | 2-6 days | 7-10 days | 11-14 days |
| 1. Feeling angry | 0-1 day | 2-6 days | 7-10 days | 11-14 days |
| 1. Finding it difficult to be around certain individuals because of the noises that they make | 0-1 day | 2-6 days | 7-10 days | 11-14 days |
| 1. Feeling irritated | 0-1 day | 2-6 days | 7-10 days | 11-14 days |
| 1. Avoiding certain situations because of the noises that you have to put up with | 0-1 day | 2-6 days | 7-10 days | 11-14 days |
| 1. Experiencing low mood because of your intolerance to certain sounds | 0-1 day | 2-6 days | 7-10 days | 11-14 days |
