# Supplementary material for: Misophonia impact questionnaire (MIQ), tinnitus impact questionnaire (TIQ), and hyperacusis impact questionnaire (HIQ): Factor analysis, test-retest reliability, and minimum detectable change using a non-clinical population
Source: PLoS One. 2025 Jun 5;20(6):e0324726. doi: 10.1371/journal.pone.0324726 (PMC12140247; doi:10.1371/journal.pone.0324726)
Supplement: S3 Appendix — (DOCX) [file pone.0324726.s003.docx]

**S3 Appendix:** Hyperacusis Impact Questionnaire

| Please answer each item to the best of your ability as close to your experience as possible.  Over the last 2 weeks, how often would you say each of the following has occurred because of certain environmental sounds that seemed too loud to you but that people around you could tolerate well? | | | | |
| --- | --- | --- | --- | --- |
| 1. Feeling anxious when hearing loud noises | 0-1 days | 2-6 days | 7-10 days | 11-14 days |
| 1. Avoiding certain places because it is too noisy | 0-1 days | 2-6 days | 7-10 days | 11-14 days |
| 1. Lack of concentration in noisy places | 0-1 days | 2-6 days | 7-10 days | 11-14 days |
| 1. Unable to relax in noisy places | 0-1 days | 2-6 days | 7-10 days | 11-14 days |
| 1. Difficulty in carrying out certain day-to-day activities/tasks in noisy places | 0-1 days | 2-6 days | 7-10 days | 11-14 days |
| 1. Lack of enjoyment from leisure activities in noisy places | 0-1 days | 2-6 days | 7-10 days | 11-14 days |
| 1. Experiencing low mood because of your intolerance to sound | 0-1 days | 2-6 days | 7-10 days | 11-14 days |
| 1. Getting tired quickly in noisy places | 0-1 days | 2-6 days | 7-10 days | 11-14 days |
