# Supplementary material for: Misophonia impact questionnaire (MIQ), tinnitus impact questionnaire (TIQ), and hyperacusis impact questionnaire (HIQ): Factor analysis, test-retest reliability, and minimum detectable change using a non-clinical population
Source: PLoS One. 2025 Jun 5;20(6):e0324726. doi: 10.1371/journal.pone.0324726 (PMC12140247; doi:10.1371/journal.pone.0324726)
Supplement: S4 Appendix — (DOCX) [file pone.0324726.s004.docx]

**S4 Appendix :** Misophonia Impact Questionnaire (parent version)

| Please answer each item to the best of your ability as close to your child’s experience as possible. Over the last 2 weeks, how often would you say the following has occurred because of your child’s intolerance to certain sounds related to eating, chewing gum, lip smacking, mouth noises, sniffling, breathing, clicking, and tapping? | | | | |
| --- | --- | --- | --- | --- |
| 1. Feeling anxious | 0-1 day | 2-6 days | 7-10 days | 11-14 days |
| 1. Unable to ignore certain sounds | 0-1 day | 2-6 days | 7-10 days | 11-14 days |
| 1. Experiencing difficulties in their relationships with family members or friends | 0-1 day | 2-6 days | 7-10 days | 11-14 days |
| 1. Feeling angry | 0-1 day | 2-6 days | 7-10 days | 11-14 days |
| 1. Finding it difficult to be around certain individuals because of the noises that they make | 0-1 day | 2-6 days | 7-10 days | 11-14 days |
| 1. Feeling irritated | 0-1 day | 2-6 days | 7-10 days | 11-14 days |
| 1. Avoiding certain situations because of the noises that they have to put up with | 0-1 day | 2-6 days | 7-10 days | 11-14 days |
| 1. Experiencing low mood because of their intolerance to certain sounds | 0-1 day | 2-6 days | 7-10 days | 11-14 days |
