# Supplementary material for: Misophonia impact questionnaire (MIQ), tinnitus impact questionnaire (TIQ), and hyperacusis impact questionnaire (HIQ): Factor analysis, test-retest reliability, and minimum detectable change using a non-clinical population
Source: PLoS One. 2025 Jun 5;20(6):e0324726. doi: 10.1371/journal.pone.0324726 (PMC12140247; doi:10.1371/journal.pone.0324726)
Supplement: S5 Appendix — (DOCX) [file pone.0324726.s005.docx]

**S5 Appendix :** Tinnitus Impact Questionnaire (parent version)

| Please answer each item to the best of your ability as close to your child’s experience as possible. Over the last 2 weeks, how often would you say the following has occurred because of your child’s tinnitus? Tinnitus is hearing a sound in the ears or head with no external source (e.g., buzzing, a high-pitched whistle, hissing...). | | | | |
| --- | --- | --- | --- | --- |
| 1. Lack of concentration | - 1. days | 2-6 days | 7-10 days | 11-14 days |
| 1. Feeling anxious | 0-1 days | 2-6 days | 7-10 days | 11-14 days |
| 1. Sleep difficulties (delay in falling asleep and/or difficulty getting back to sleep if woken up during the night) | 0-1 nights | 2-6 nights | 7-10 nights | 11-14 nights |
| 1. Lack of enjoyment from leisure activities | 0-1 days | 2-6 days | 7-10 days | 11-14 days |
| 1. Inability to perform certain day-to-day activities/tasks | 0-1 days | 2-6 days | 7-10 days | 11-14 days |
| 1. Feeling irritable | 0-1 days | 2-6 days | 7-10 days | 11-14 days |
| 1. Low mood | 0-1 days | 2-6 days | 7-10 days | 11-14 days |
